# Renal and Neurologic Effects of Cadmium, Lead, Mercury, and Arsenic in Children: Evidence of Early Effects and Multiple Interactions at Environmental Exposure Levels

**DOI:** 10.1289/ehp.8202

**Published:** 2005-10-20

**Authors:** Claire de Burbure, Jean-Pierre Buchet, Ariane Leroyer, Catherine Nisse, Jean-Marie Haguenoer, Antonio Mutti, Zdenek Smerhovský, Miroslav Cikrt, Malgorzata Trzcinka-Ochocka, Grazyna Razniewska, Marek Jakubowski, Alfred Bernard

**Affiliations:** 1 Unit of Industrial Toxicology and Occupational Medicine, Faculty of Medicine, Catholic University of Louvain, Belgium; 2 Centre de recherches en santé-travail-ergonomie-Laboratoire Universitaire de Médecine du Travail, Université Lille 2, Lille, France; 3 Laboratorio di Tossicologia Industriale, Universita degli Studi di Parma, Parma, Italy; 4 National Institute of Public Health, Prague, Czech Republic; 5 Nofer Institute of Occupational Medicine, Lodz, Poland

**Keywords:** arsenic, biomarkers, cadmium, dopaminergic, heavy metals, interactions, lead, mercury, renal

## Abstract

Lead, cadmium, mercury, and arsenic are common environmental pollutants in industrialized countries, but their combined impact on children’s health is little known. We studied their effects on two main targets, the renal and dopaminergic systems, in > 800 children during a cross-sectional European survey. Control and exposed children were recruited from those living around historical nonferrous smelters in France, the Czech Republic, and Poland. Children provided blood and urine samples for the determination of the metals and sensitive renal or neurologic biomarkers. Serum concentrations of creatinine, cystatin C, and β_2_-microglobulin were negatively correlated with blood lead levels (PbB), suggesting an early renal hyperfiltration that averaged 7% in the upper quartile of PbB levels (> 55 μg/L; mean, 78.4 μg/L). The urinary excretion of retinol-binding protein, Clara cell protein, and *N*-acetyl-β-d-glucosaminidase was associated mainly with cadmium levels in blood or urine and with urinary mercury. All four metals influenced the dopaminergic markers serum prolactin and urinary homovanillic acid, with complex interactions brought to light. Heavy metals polluting the environment can cause subtle effects on children’s renal and dopaminergic systems without clear evidence of a threshold, which reinforces the need to control and regulate potential sources of contamination by heavy metals.

Environmental pollution of industrialized countries by heavy metals such as lead, cadmium, mercury, and the metalloid arsenic is largely the consequence of past emissions by nonferrous industries. Although stringent measures and controls have been put into place during the last decades, high levels of these pollutants still persist in the soils and sediments—and therefore also in the food chain—with possible consequences of chronic environmental exposure of the populations living in those areas. Moreover, natural contamination such as geologic arsenic or lifestyle-related factors such as the inorganic mercury in dental amalgam can further contribute to increase the burden of human exposure to these toxicants.

Most of our knowledge concerning the health effects of toxic metals largely stems from studies conducted on populations with relatively high exposure usually to individual metals in industry or in heavily polluted environments. Very few studies have addressed the possible effects of chronic low environmental exposure to mixtures of these metals, particularly with regard to their possible interactions, although this is precisely the situation most commonly encountered by the general population of industrialized countries. Furthermore, there is a definite paucity of data concerning children, a specific cause for concern because children are known to absorb metals more readily than adults and are particularly sensitive for biologic and developmental reasons (Fels et al. 1998).

Among possible target organs of heavy metals, the kidney and central nervous system appear to be the most sensitive ones. Inorganic heavy metals have been known for a long time to be nephrotoxic at relatively high levels of exposure, with numerous reports of tubulointerstitial nephritis possibly leading to renal failure, in most cases linked to high occupational or environmental exposure (Fowler 1996). Early signs of renal dysfunction can, however, be found with exposure to low environmental levels of these heavy metals, consisting in decreased glomerular filtration rate (GFR) (lead) or increased urinary loss of tubular enzymes (cadmium). These effects have been described mainly in adults, but certain reports have also shown them to occur in children (Bernard et al. 1995; Verberk et al. 1996). Neurotoxic effects of heavy metals are also well documented, especially for mercury and lead, with numerous reports of neurobehavioral changes after occupational exposure and of developmental effects in children with pre- or early postnatal exposure (Davidson et al. 2004; Lidsky and Schneider 2003). However, experimental studies suggest that other metals such as cadmium and arsenic could also interfere with the nervous system and that all four metals may influence the dopaminergic system in different ways (Lafuente et al. 2003b; Pohl et al. 2003). There is, however, a need to elucidate which exposure levels are likely to cause these biologic effects, particularly in children, and to what extent the four metals could interfere and interact in mixed exposures.

To address some of these issues, in the present study we focused on populations of children living in three separate European regions known for their historical levels of pollution in France, Poland, and the Czech Republic. The levels of exposure to cadmium, lead, mercury, and arsenic were determined in about 800 children together with a set of sensitive biomarkers of kidney function and of the dopaminergic system.

## Materials and Methods

### Studied European areas.

#### France.

The environmentally exposed area studied concerned 10 municipalities in the Nord-Pas-de-Calais located in an 8-km radius around both a zinc smelter (near the city of Auby) and a lead and zinc smelter (near the city of Noyelles-Godault) 3.5 km apart. The foundries had both been operational since the second half of the 19th century and had liberated vast quantities of heavy metals in the atmosphere until 1975, and then gradually reduced their emissions by more than 90% to slightly more than 24 tons of lead and 950 kg of cadmium in 1996; the Noyelles-Godault smelter closed down in 2003. Soil contamination varied between 100 and 1,700 ppm for lead (values > 1,000 ppm in a 500-m radius around the foundries), between 0.7 and 233 ppm cadmium, and between 101 and 22,257 ppm zinc, the highest values being found within 500 m of the smelters. Lead and cadmium level determinants were mainly linked to habitat distance from the factories, drinking tap water, and, for cadmium, consumption of local produce, fish, and crustaceans (Leroyer et al. 2000, 2001). The French control area concerned 20 municipalities of the same region that were unpolluted by heavy metals.

#### Czech Republic.

The environmentally polluted area studied was centered around the historic site of Pribram, known for its mining since the 10th century. Indeed, silver, lead, and other precious metals extracted in that area represented, at the end of the 19th century, 97.7% of the total Austro-Hungarian production. Uranium mining also appeared in the 20th century but ceased in 1991; the metal mines of Pribram ceased ore mining in 1979. The lead smelter examined in our study emitted > 250 metric tons of lead per year into the atmosphere until 1982, when filters were installed, reducing emissions to < 20 metric tons per year (Bernard et al. 1995). Soil lead levels in 1994 varied between 100 and > 5,000 ppm (values > 1,000 ppm within 1,000 m of the smelter). The control area was in a nonpolluted rural area, Sedlcany, located east of Pribram.

#### Poland.

The environmentally polluted area studied consisted of small villages located within a 10-km radius around the copper mills of Legnica, where a previous study concerning children had indicated that 22% of children had blood lead levels (PbB) > 100 μg/L (Jakubowski et al. 1996). The control area was in a nonpolluted rural region free of heavy industry, Gorzow, in northwest Poland.

### Studied population.

After protocol approval of the study by the local ethical committees, a total of 804 children 8.5–12.3 years of age from France, Poland, and the Czech Republic took part in the study: 400 French children (200 boys: 101 exposed, 99 controls; 200 girls: 99 exposed, 101 controls), 215 Polish children (99 boys: 50 exposed, 49 controls; 116 girls: 59 exposed, 57 controls), and 189 Czech children (97 boys: 49 exposed, 48 controls; 92 girls: 45 exposed, 47 controls). Exposed children had lived at least 8 years near non-ferrous smelters, whereas their controls were recruited from areas unpolluted by heavy metals in the same region of each country. Children were recruited on a volunteer basis with letters sent via schools to their parents, explaining the objectives and protocol of the study as well as the selection criteria (no diabetes or renal disease and, for girls, absence of menses). We considered as volunteers only children meeting these criteria and whose parents had given their written permission for examination of their child and sampling of blood and urine. Lead concentrations in polluted soils were, on average, > 200 ppm, with values > 1,000 ppm in the immediate vicinity of the factories. The study protocol was approved by local ethical committees and complied with all applicable requirements of U.S. and/or international regulations.

### Biologic samples.

Biologic samples were collected with the written permission of either the children’s parents or the person responsible for them. Blood samples were collected and centrifuged, and 2 mL of serum were stored at –80°C until analysis. The usual precautions were taken to avoid external contamination during collection, storage, and processing of samples by checking that all containers were metal-free. Untimed urine samples were collected during daytime and stored at –20°C. Because of the small volumes of some samples, all biologic parameters could not be determined in all subjects; the exact numbers that could be analyzed are indicated in the tables.

### Analyses.

All analyses of renal and dopaminergic biomarkers were performed under similar experimental conditions in the same laboratory (Brussels) within 6 months of collection. In contrast, metals were analyzed in each country using methods that were standardized and controlled at the beginning of the project. Standardization involved the combined analysis of the initial 10% of all samples, performed by all partners, and the results were judged satisfactory according to the criteria of Bland and Altman (1986). We conducted the common analyses for all three cohorts as follows: Serum creatinine (CreatS) and urinary creatinine (CreatU) were measured by the methods of Heinegard and Tiderstrom, and Jaffé, respectively (Price et al. 1996); serum cystatin C (CystCS), serum β_2_-microglobulin (B2MS), and urinary retinol-binding protein (RBPU) were quantified by automated latex immunoassays (Bernard et al. 1981). The total activity of *N*-acetyl-β-d-glucosaminidase in urine (NAGTU) was determined colorimetrically using a NAG kit (PPR Diagnostics Ltd., London, UK), as described elsewhere (Price et al. 1996). These renal markers were selected because they are known to be among the most sensitive and reliable indicators for screening renal damage in populations that are occupationally or environmentally exposed to heavy metals (Bernard and Hermans 1997). Urinary homovanillic acid (HVAU), one of the end-products of dopamine metabolism, was assayed in urine using isocratic HPLC, whereas serum prolactin (PRLS), whose secretion is under control of the dopaminergic system, was measured in serum by chemiluminescent enzyme immunoassay, as previously described (Alvarez Leite et al. 2002). We asssessed heavy metal exposure by measuring by atomic absorption spectrometry for PbB, whole-blood cadmium (CdB), urinary cadmium (CdU), and urinary mercury (HgU) as described previously (de Burbure et al. 2003; Leroyer et al. 2000, 2001). Urinary arsenic levels (AsU) were studied only in Polish and Czech children and were measured after arsine generation as the sum of inorganic arsenic and its methylated metabolites (monomethylarsonic acid, dimethylarsinic acid) without notable interference by seafood trimethylated arsenicals (Buchet et al. 2003). All urinary parameter assays were adjusted for CreatU. Urine samples with a creatinine concentration < 0.3 or > 3.0 g/L (81 children; range, 0.17–3.07 g/L) were excluded from the data analyses.

### Statistical analysis.

We used categorical variables for sex and area of residence, and all continuous variables except age were normalized by log-transformation before statistical analysis. Parameter distribution normality was assessed with the Shapiro-Wilk test. The logarithmic transformation was satisfactory for body mass index (BMI), PbB, CdU, CreatS, CreatU, serum Clara cell protein (CC16S), urinary Clara cell protein (CC16U), CystCS, B2MS, PRLS, NAGTU, and RBPU. We calculated normal rank values according to the Blom procedure for CdB, HgU, and HVAU. We compared group means by the unpaired Student’s *t*-test or, in cases of more than two groups, by Duncan test after analysis of variance (ANOVA). In addition, we analyzed the influence of sex, exposure, and their possible interaction by two-way ANOVA for each country. Determinants of renal and dopaminergic parameters studied were traced by stepwise multiple regression analysis using as independent variables log PbB, rank CdB, rank HgU, log AsU where applicable (Czech and Polish), log CreatU, log BMI, age, sex, and area of residence, as well as all first-order metal interaction terms. Polish and Czech children were assessed both separately (French children were studied previously: de Burbure et al. 2003; Leroyer et al. 2000, 2001) and together to study the impact of arsenic. All multiple regression analyses were conducted each time twice, considering either CdB or CdU as the cadmium exposure indicator. Although all urinary parameters were adjusted for CreatU, we performed multiple regression analyses by again testing CreatU levels to eliminate any residual effect of the diuresis. Stepwise multiple regression analyses used a *p*-level equal to 0.25 for entry and a level of 0.05 for staying in the model. The level of statistical significance was set at *p* < 0.05. To illustrate the relationships between some parameters and a specific element, we used equations describing the multiple regression models to adjust the dependent variable for the mean value covariates included in the model (when necessary, female sex and a CreatU of 1 g/L were selected). The means of these corrected values in groups according to four ranges of increasing values of the specific element (quartiles) were then compared. Where parameter values were corrected for determinants other than a single metal exposure parameter, the latter was considered in quartiles to allow ANOVA and testing of the significance of differences between mean values. The consideration of quartiles of a second element in each quartile of a first one helped to illustrate the presence of interactions between elements. When an element influenced a studied parameter both alone and in an interaction with another one, the total population of children was divided into quartiles according to the first element, and each of these was divided into quartiles according to the element playing in interaction only. We used the statistical package SAS (version 6; Cary, NC, USA) (particularly the univariate, GLM, and REG procedures) for all statistical analyses.

## Results

Table 1 shows the mean values of all biologic parameters between control and exposed children separated by sex and country. As expected, both boys and girls of all three countries had significantly higher levels of PbB and CdB in exposed areas compared with their respective controls. There was no significant difference in HgU levels between exposed and control children in France or Poland. Czech children in the polluted area actually had lower HgU and AsU levels than did their controls, whose AsU levels were unexpectedly almost double those of other cohorts. Comparison of metal levels in the different countries revealed that French children in both the exposed and control areas had significantly higher levels of HgU, CdB, and CdU than did Czech and Polish children, whereas Polish children from the exposed area had significantly higher PbB levels than did all the others. In each country, the influence of sex and its interaction with exposure was studied by a two-way ANOVA. Boys had significantly higher PbB levels than girls, but no sex-related difference was observed for the three other metals. As expected, girls had higher PRLS and lower CreatS compared with boys, both significantly so in France and Poland. No interaction between sex and exposure was found in most biomarkers except in some groups for CC16U and CystCS.

We conducted multiple regression analyses taking sex, CreatU, the levels of all four metals, and their first-order interactions as independent variables on the whole population, taking two models, with either CdB or CdU, into consideration (Table 2). Remarkably, the three markers of GFR—CreatS, CystCS, and B2MS—were negatively correlated with PbB levels in both models. CystCS was the only parameter not influenced by any other determinant. CreatS correlated also negatively with CdU, and both models evidenced an interaction between PbB and HgU increasing CreatS. B2MS and CC16S, on the other hand, were also negatively correlated with HgU. RBPU and CC16U showed a significant positive correlation with both CdB and CdU levels, whereas NAGTU increased significantly with both CdU and CdB and with HgU levels in both models. Dopaminergic markers indicated a decrease in PRLS and a corresponding increase in HVAU with rising CdB, CdU, and HgU. Removing CreatU from the independent variables (used to eliminate any residual influence of diuresis) did not alter the above results but revealed an added negative correlation of B2MS with CdB (data not shown). In the combined Czech and Polish populations, AsU was found to be a positive determinant of CC16U and as an interactive term, modulating several of the associations with PbB, CdB, CdU, and HgU indicated above (data not shown).

**Figure 1:**
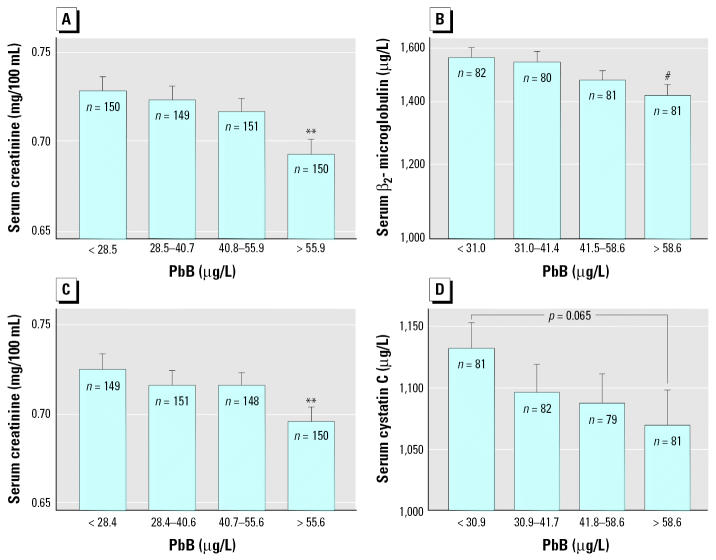
Mean concentrations of CreatS, B2MS, and CystCS in the total population of children divided in quartiles of PbB, after standardization for other cofactors. Variables preceded by R are ranked variables. (*A*) CreatS after standardization for CreatU, sex, RCdB × RHgU, and PbB × RHgU interactions. (*B*) B2MS after standardization for RHgU and CreatU. (*C*) CreatS after standardization for CdU, CreatU, sex, and PbB × RHgU interaction. (*D*) CystatinCS. Error bars denote SE. Statistically significant difference from first quartile: **p < 0.01; ^#^*p* < 0.001.

**Figure 2:**
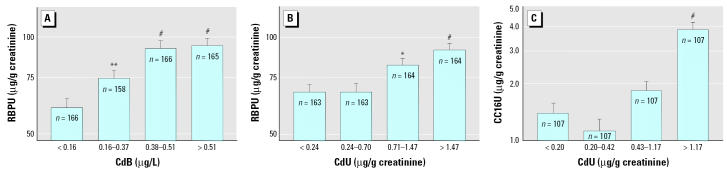
Mean RBPU and CC16U in the total population of children divided in quartiles of CdB or CdU, after standardization for other cofactors. Variables preceded by R are ranked variables. (*A*) RBPU after standardization for CreatU, RCdB × RHgU, and PbB × RHgU interactions. (*B*) RBPU after standardization for CreatU and PbB × CdU interaction. (*C*) CC16U after standardization for CreatU. Error bars denote SE. Statistically significant difference from first quartile: *p < 0.05; **p < 0.01; ^#^*p* < 0.001.

**Figure 3:**
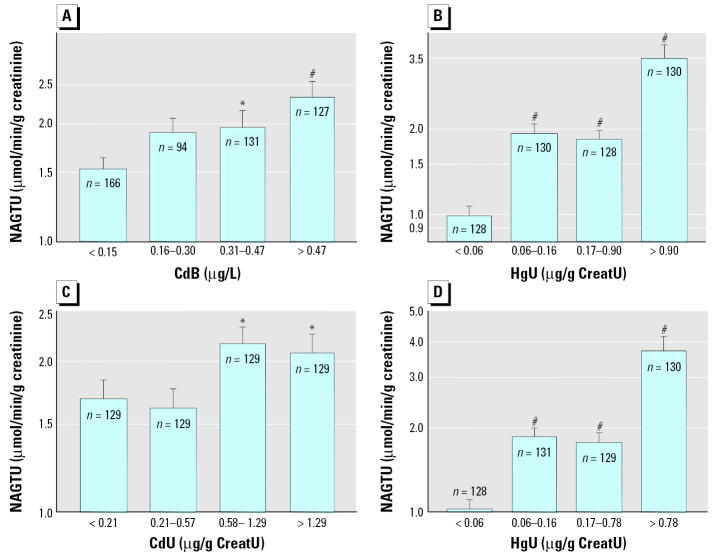
Mean concentrations of NAGTU in the total population of children divided in quartiles of CdB, CdU, or HgU after standardization for other cofactors, considering either CdB or CdU as independent variable. Variables preceded by R are ranked variables. (*A*) NAGTU after standardization for RHgU, CreatU, RHgU × RCdB, and PbB × RHgU interactions. (*B*) NAGTU after standardization for RCdB, CreatU, RHgU × RCdB, and PbB × RHgU interactions. (*C*) NAGTU after standardization for RHgU, CreatU, and PbB × RHgU interactions. (*D*) NAGTU after standardization for CdU, CreatU, and PbB × RHgU interactions. Error bars denote SE. Statistically significant difference from first quartile: *p < 0.05; ^#^*p* < 0.001.

**Figure 4:**
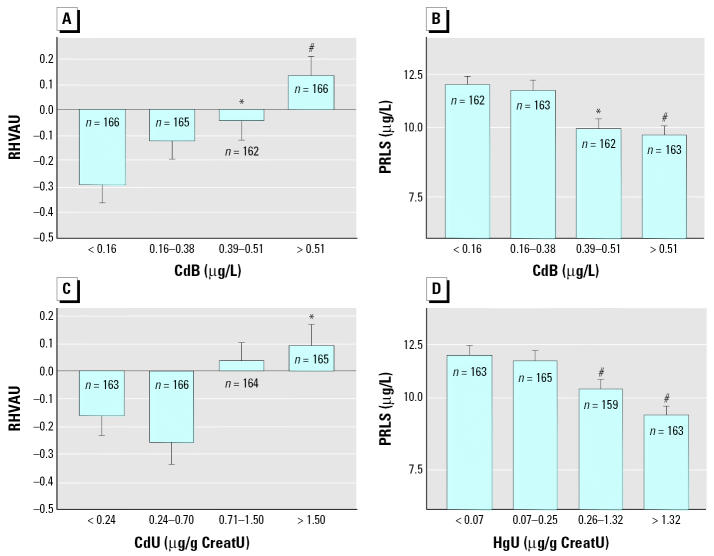
Mean concentrations of ranked HVAU and PRLS in the total population of children divided in quartiles of CdB, CdU, or HgU after standardization for other cofactors, considering either CdB or CdU as independent variable. Variables preceded by R are ranked variables. (*A*) RHVAU after standardization for CreatU, RCdB × RHgU, and PbB × RHgU interactions. (*B*) PRLS after standardization for CreatU, sex, and RHgU. (*C*) RHVAU after standardization for CreatU and PbB × RHgU interaction. (*D*) PRLS after standardization for RCdB, sex, and CreatU. Error bars denote SE. Statistically significant difference from first quartile: *p < 0.05; ^#^*p* < 0.001.

We assessed dose–effect relationships by dividing the children in quartiles of increasing levels of the metals in urine or blood and comparing by ANOVA the values of renal or neurologic biomarkers adjusted for other covariates. As illustrated in Figure 1, levels of CreatU, CystCS, and B2MS decreased in a dose-dependent way with increasing PbB, with an apparent threshold around 50 μg/L PbB, where statistical significance was reached. The increased RBPU or CC16U was also closely related to the internal dose of cadmium, with no detectable threshold in the case CdB and a threshold around 1 μg/g creatinine for CdU (Figure 2). A similar pattern emerged for the increased NAGTU with cadmium exposure (Figure 3), indicating also a very low threshold for both CdB (0.31 μg/L) and CdU (0.58 μg/g creatinine). NAGTU increased with HgU from very low concentrations, as low as 0.06 μg/g creatinine, when adjusting for CdB or CdU (Figure 3). As shown in Figure 4, similar dose–effect relationships with no detectable or very low thresholds were also observed with dopaminergic markers, confirming the decrease in PRLS and increase in HVAU with rising CdB, CdU, and HgU.

**Figure 5:**
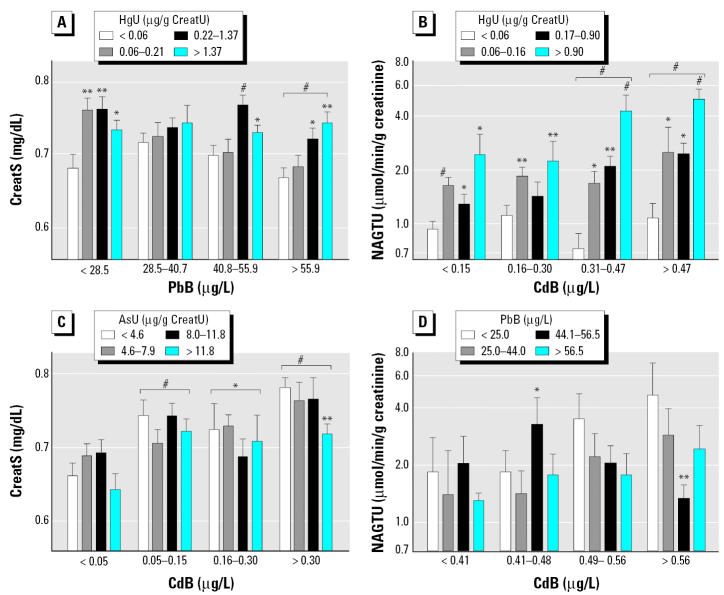
Mean concentrations of CreatS or NAGTU in children divided in quartiles of PbB or CdB concentrations and further subdivided in quartiles of AsU or HgU. Variables preceded by R are ranked variables. (*A*) Whole population (*n* = 600) after standardization for CreatU, sex, and RCdB × RHgU interaction. (*B*) Whole population (*n* = 518) after standardization for CreatU and LPbB × RHgU interaction. (*C*) Czech and Polish children (*n* = 320) after standardization for CreatU, sex, LPbB, and LAsU × RHgU interaction. (*D*) French children (*n* = 195) after standardization for CreatU. Error bars denote SE. Statistically significant difference from the first quartiles: *p < 0.05; **p < 0.01; ^#^*p* < 0.001.

The most interesting metal interactions with regard to renal biomarkers are illustrated in Figure 5. In particular, it can be seen that HgU inhibits the PbB-related renal hyper-filtration (i.e., the PbB-related decrease in CreatS), whereas it potentiates the increased NAGTU linked to CdB. By contrast, AsU appears to inhibit the increase in CreatS associated with CdB, and PbB tends to antagonize the CdB-related rise in NAGTU. With regard to dopaminergic markers (data not shown), PbB appears to antagonize the significant HgU-related decrease in HVAU, whereas HgU exacerbates the increase in HVAU linked to CdB.

## Discussion

Although children living around nonferrous smelters were significantly more exposed to lead and cadmium than were their controls, the mean levels of lead, cadmium, mercury, and arsenic in blood or urine of all studied groups were well within the range of values normally found in the European population, including children, as described in other European studies (Camerino et al. 2002; Hotz et al. 1999; Staessen et al. 2001). Even the higher mean PbB levels observed in Pribram (Czech Republic) were noticeably lower (by half) than those described in that same area about 10 years before the present study (Bernard et al. 1995). It was, however, clear that the children of the three countries, albeit selected by means of identical criteria, varied significantly with regard to the metal baseline levels as observed in control cohorts. These variations most probably reflect differences in the environmental levels of these metals as well as in the lifestyle of these children, in particular, their dietary habits, home environment, and dental care. The 2-fold increase in AsU levels observed in the Czech control children was nevertheless an unexpected finding, eventually linked to high arsenic levels in the local underground water, probably because of known gold deposits in the region.

The most interesting and consistent finding evidenced by our study in children was an overall inverse relationship between CreatS, B2MS, CystCS, and PbB, suggesting that environmental lead induces an early renal hyperfiltration, similar to that described in experimental animals with lead-induced renal cortex hypertrophy (Khalil-Manesh et al. 1992). The increase in GFR induced by lead can be estimated using equations relating the GFR to CreatS, CystCS, and B2MS (Donadio et al. 2001; Risch et al. 1999). Depending on the serum marker used, the increase in GFR ranged from 7 to 11% in children in the upper PbB quartile (> 55 μg/L; mean PbB, 78.4 μg/L). Renal hyperfiltration had already been described in lead smelter workers with much higher PbB levels (Roels et al. 1994; Weaver et al. 2003a) but not yet in a general population with low environmental exposure, and never to our knowledge in children. One explanation brought forward for these observations comes in part from the hyperfiltration theory, described as a paradoxical increase in GFR linked to altered glomerular hemodynamics (Weaver et al. 2003a). According to recent experimental studies, the initial mechanism may well depend on lead-induced production of reactive oxygen species up-regulating cyclooxygenase (COX-2) expression in the vascular smooth muscle wall (Courtois et al. 2003). These findings would also tie in with those in workers with lead-induced renal hyperfiltration who show a decreased production of prostaglandin (PGF2) and an increased production of thromboxane (Roels et al. 1994). Moreover, this explanation is supported by recent findings in lead-exposed workers showing in addition that the renal response to lead, including hyperfiltration, was modulated by genetic polymorphisms in δ-aminolevulinic acid dehydratase (*ALAD 2* allele or *ALAD 1-2* genotype) and nitric oxide synthase (*eNOS* variant allele) genes (Weaver et al. 2003b). Interestingly, although CystCS was affected only by PbB levels, both CreatS and B2MS were also found to correlate with cadmium or mercury independently or in interaction with PbB. These relationships could be explained by various competitive interactions between metals for intracellular binding sites, causing, for instance, a displacement of lead from its renal store, as has been shown with both cadmium and mercury (Fowler 1998).

With regard to tubular effects, the most interesting effects were consistent increases in the RBPU, CC16U, and NAGTU in correlation to cadmium levels. Of note, these increases were found with both CdB and CdU, thus excluding the possibility of secondary associations due to the dependence of the urinary excretion of proteins and cadmium on the integrity of renal function. These findings provide further evidence that environmental cadmium, even at currently observed levels, can affect the renal tubules of children. Interestingly, urinary mercury levels were found to correlate with some tubular markers, both in interactions (CC16U and RBPU) and independently of other metals (NAGTU). To our knowledge, there are no reports showing evidence of tubular dysfunction at such low levels of HgU. What is particularly disturbing with these tubular effects is the very low threshold of metal exposure from which they become statistically significant. For instance, RBPU and CC16U were observed to increase significantly from mean CdU (< 1 μg/g creatinine) and CdB (< 0.5 μg/L) that are in the range of mean values currently observed in most industrialized countries. These thresholds are five to ten times lower than those established in adult populations living in heavily polluted environments, such as in China or Japan, suggesting that children’s kidneys could be much more sensitive to heavy metals than those of adults.

An important issue to bear in mind in the interpretation of our data is that the renal effects observed in this study could reflect an early renal response to metals that could be purely adaptative and/or reversible depending on the type of metal and the studied end point (Roels et al. 1997). Renal hyperfiltration has commonly been observed in various renal diseases and clinical conditions such as early type I diabetes, sickle cell disease, obesity, and high-protein diet; but in most cases it is associated with clinical anomalies such as hypertension, and it is much more pronounced than that observed in the present study (Courtois et al. 2003; Friedman 2004). The small lead-related renal hyperfiltration observed in our study could merely reflect hemodynamic changes due to an interference of lead with prostaglandin metabolism, which quite conceivably could be transient and entirely reversible. Similarly, the preclinical tubular effects associated mainly with cadmium and mercury could also be the manifestation of proximal tubular alterations, which could also be reversible if one refers to observations made in adults with incipient cadmium nephropathy (Roels et al. 1997). However, given the very few studies performed in children, it is difficult to assess the actual biologic and clinical significance of these early renal changes in children. It cannot be excluded that these effects could be potentially adverse, rendering, for instance, the kidneys more sensitive to other stressors later in life.

Contrary to findings in lead-exposed workers (Govoni et al. 1987; Lucchini et al. 2000), lead did not appear to increase PRLS in the various children populations. CdB and HgU, by contrast, were both negatively correlated with PRLS but correlated positively with HVAU. These correlations, which indicate an increased dopamine metabolism, agree with recent experimental data in rats, showing that cadmium interferes with biogenic amine release from the hypothalamus, thereby inhibiting prolactin secretion (Lafuente et al. 2003a), whereas inorganic mercury stimulates increased striatal dopamine levels (Faro et al. 2003). These observations are also consistent with recent results in adults occupationally exposed to inorganic mercury (Carta et al. 2003). Arsenic, except for its interactive effects, did not directly influence either PRLS or HVAU, contrary to experimental evidence, indicating that effects reported in animals do not occur at low environmental exposure levels (Delgado et al. 2000; Rodriguez et al. 1998).

In conclusion, our data show that heavy metals polluting the environment can cause subtle effects on the children’s renal and dopaminergic systems. In particular, renal hyperfiltration appears an early response to lead, whereas cadmium exposure is associated with subtle tubular effects modulated by coexposure to mercury and lead. These findings at current low environmental exposure levels, sometimes with no detectable threshold, reinforce the need to control and regulate potential sources of contamination by heavy metals.

## Figures and Tables

**Table 1:** Parameters studied according to country, sex , and level of exposure of children.

	Female				Male				
	Control		Exposed		Control		Exposed		
Parameter	No.	Mean (GSD)^a^	No.	Mean (GSD)^a^	No.	Mean (GSD)^a^	No.	Mean (GSD)^a^	Two-way ANOVA *p* < 0.05
French children									
Age (years)	94	10.1 ± 0.7	94	10.1 ± 0.7	94	10.3 ± 0.7	100	10.1 ± 0.7	
BMI (kg/m^2^)	94	17.1 (1.18)	94	17.7 (1.17)	94	17.1 (1.13)	100	17.4 (1.16)	
PbB (μg/L)	94	28.1 (1.93)	94	36.4 (1.75)*	94	34.2 (1.93)	100	42.1 (2.02)*	Exposure; sex
CdB (μg/L)	92	0.47 (1.38)	93	0.5 (1.25)	93	0.46 (1.40)	100	0.52 (1.40)*	Exposure
CdU (μg/g cr)	94	0.91 (2.74)	93	1.07 (2.86)	93	1.02 (3.05)	97	1.15 (3.10)	
HgU (μg/g cr)	78	0.89 (5.20)	87	1.19 (5.78)	85	0.99 (4.93)	89	0.92 (6.60)	
CreatS (mg/L)	77	7.0 (1.14)	77	7.2 (1.15)	79	7.3 (1.14)	89	7.5 (1.13)	Sex
CC16U (μg/g cr)	24	7.72 (2.61)	29	3.88 (1.95)*	25	5.08 (2.44)	35	5.57 (2.43)	Interaction
NAGTU (UI/g cr)	55	2.49 (3.77)	59	1.71 (2.21)	53	2.29 (4.15)	54	2.15 (2.66)	
RBPU (μg/g cr)	92	108.0 (1.81)	93	107.7 (1.81)	93	94.3 (1.79)	98	97.6 (1.83)	
PRLS (μg/L)	91	8.53 (1.79)	90	9.36 (1.71)	92	7.35 (1.68)	100	8.19 (1.72)	
HVAU (mg/g cr)	94	5.23 (1.69)	94	5.69 (1.76)	94	5.59 (1.62)	100	5.29 (1.61)	Sex
Polish children									
Age (years)	50	10.3 ± 0.8	50	10.2 ± 0.8	35	10.1 ± 0.8	44	10.0 ± 0.9	
BMI (kg/m^2^)	50	16.5 (1.16)	50	16.3 (1.12)	35	17.9 (1.20)	44	16.8 (1.14)	
PbB (μg/L)	50	34.2 (1.39)	47	57.2 (1.51)*	35	38.1 (1.45)	42	65.1 (1.65)*	Exposure; sex
CdB (μg/L)	50	0.08 (2.25)	45	0.19 (2.58)*	35	0.07 (1.85)	42	0.19 (2.36)*	Exposure
CdU (μg/g cr)	50	0.45 (2.52)	49	0.56 (3.24)	35	0.44 (4.13)	44	0.68 (3.08)	
HgU (μg/g cr)	50	0.05 (1.59)	50	0.06 (1.53)*	34	0.06 (1.51)	43	0.06 (1.61)	
AsU (μg/g cr)	50	5.99 (1.93)	49	7.98 (1.80)*	34	6.73 (2.08)	44	8.74 (1.77)	Exposure
CreatS (mg/L)	50	6.7 (1.12)	49	6.1 (1.17)*	35	6.9 (1.11)	43	0.66 (1.11)	Exposure; sex
CystCS (μg/L)	50	1190 (1.20)	50	1035 (1.28)*	34	1105 (1.25)	44	1100 (1.26)	Interaction
B2MS (μg/L)	50	1710 (1.20)	50	1370 (1.34)*	34	1810 (1.20)	44	1450 (1.29)*	Exposure
CC16S (μg/L)	50	13.5 (1.57)	50	12.2 (1.39)	34	13.7 (1.68)	44	11.7 (1.60)	
CC16U (μg/g cr)	50	2.14 (1.96)	50	1.78 (2.42)	34	3.46 (2.96)	43	2.33 (2.43)*	Exposure; sex
NAGTU (UI/g cr)	50	2.79 (2.08)	50	2.80 (1.96)	34	2.93 (2.06)	43	2.71 (1.94)	
RBPU (μg/g cr)	50	90.8 (1.80)	50	55.40 (2.16)*	34	92.00 (1.83)	43	61.2 (2.31)*	Exposure
PRLS (μg/L)	48	13.1 (1.40)	48	14.7 (1.54)	35	10.5 (1.59)	43	11.7 (1.59)	
HVAU (mg/g cr)	50	6.81 (1.50)	50	7.45 (1.26)	35	6.96 (1.41)	44	6.79 (1.28)	Sex
Czech children									
Age (years)	36	9.4 ± 1.3	39	9.5 ± 1.1	43	9.4 ± 0.8	44	9.4 ± 1.1	
BMI (kg/m^2^)	36	17.9 (1.17)	38	16.76 (1.14)	43	16.80 (1.15)	42	16.50 (1.12)	
PbB (μg/L)	36	34.00 (1.35)	39	40.57 (1.51)*	43	36.10 (1.37)	42	49.90 (1.47)*	Exposure; sex
CdB (μg/L)	36	0.20 (1.43)	39	0.24 (1.62)	43	0.20 (1.47)	42	0.29 (1.73)*	Exposure
CdU (μg/g cr)	36	0.22 (1.66)	39	0.25 (1.89)	43	0.22 (1.51)	44	0.24 (1.63)	
HgU (μg/g cr)	35	0.32 (3.14)	39	0.18 (2.50)*	43	0.26 (2.81)	44	0.13 (3.59)*	Exposure
AsU (μg/g cr)	36	11.00 (1.75)	37	5.26 (2.07)*	43	13.40 (1.85)	43	5.32 (1.78)*	Exposure
CreatS (mg/L)	35	7.9 (1.11)	37	7.9 (1.11)	43	8.0 (1.10)	43	8.2 (1.11)	
CystCS (μg/L)	35	1114 (1.19)	37	1114 (1.19)	43	1068 (1.17)	43	1050 (0.91)	
B2MS (μg/L)	35	1432 (1.18)	37	1495 (1.19)	43	1424 (1.22)	43	1534 (1.20)	
CC16S (μg/L)	35	8.47 (1.58)	37	10.18 (1.49)	43	9.72 (1.54)	43	10.10 (1.44)	
CC16U (μg/L)	36	1.09 (3.76)	39	0.46 (4.14)*	43	0.89 (3.11)	44	1.13 (3.31)	Interaction
NAGTU (UI/g cr)	36	1.31 (2.08)	39	1.78 (2.04)	43	1.30 (2.50)	44	1.43 (2.40)	
RBPU (μg/g cr)	36	49.50 (2.17)	39	51.30 (2.06)	43	44.9 (1.99)	44	44.40 (1.98)	
PRLS (μg/L)	35	15.60 (1.62)	37	11.00 (1.40)	43	13.40 (1.44)	43	11.1 (1.39)	Exposure
HVAU (mg/g cr)	35	3.00 (2.72)	39	4.78 (1.54)*	43	3.46 (1.90)	44	4.70 (1.80)*	Exposure

cr, creatinine.

a Data are geometric means (GSD), except for age (arithmetic mean ± SD). Statistically significant difference between control and exposed children:

* *p* < 0.05. Effects of sex and exposure and their interaction were assessed by two-way ANOVA.

**Table 2:** Multiple regression analysis of the determinants of renal and neurologic biomarkers in the whole population of children.

	Models with CdB				Models with CdU			
Dependent variable	Independent variable	Regression coefficient	Partial *r*^2^	*p*-Value	Independent variable	Regression coefficient	Partial *r*^2^	*p*-Value
Serum markers								
LCreatS	LPbB	–0.026	0.012	0.007	LPbB	–0.023	0.013	0.02
	LCreatU	0.079	0.043	< 0.001	LCreatU	0.069	0.029	< 0.001
	Sex	0.022	0.032	< 0.001	Sex	0.022	0.032	< 0.001
	RHgU × RCdB	–0.008	0.011	0.004	LCdU	–0.030	0.046	< 0.001
	LPbB × RHgU	0.006	0.022	< 0.001	LPbB × RHgU	0.006	0.036	< 0.001
LCystCS	LPbB	–0.056	0.016	0.02	LPbB	–0.053	0.015	0.03
LB2MS	LPbB	–0.095	0.023	0.01	LPbB	–0.100	0.025	0.004
	RHgU	–0.023	0.028	0.02	RHgU	–0.024	0.028	0.002
	LCreatU	–0.070	0.018	0.02	LCreatU	–0.076	0.021	0.008
LPRLS	RCdB	–0.032	0.048	< 0.001	LPbB × LCdU	–0.052	0.033	< 0.001
	RHgU	–0.038	0.012	0.004	RHgU	–0.042	0.038	< 0.001
	LCreatU	–0.149	0.015	< 0.001	LCreatU	–0.191	0.019	< 0.001
	Sex	–0.063	0.019	< 0.001	Sex	–0.062	0.018	< 0.001
Urinary markers								
LCC16U	LCreatU	–0.625	0.059	< 0.001	LCreatU	–0.432	0.023	< 0.001
	LPbB × RCdB	0.055	0.023	< 0.001	LCdU	0.359	0.134	< 0.001
	RHgU × RCdB	0.131	0.049	< 0.001				
LRBPU	RCdB	0.060	0.049	< 0.001	LCdU	0.097	0.054	< 0.001
	LCreatU	–0.322	0.055	< 0.001	LCreatU	–0.243	0.036	< 0.001
	LPbB × RHgU	0.027	0.013	< 0.001	LPbB × RHgU	0.039	0.033	< 0.001
	RHgU × RCdB	0.040	0.017	0.002				
LNAGTU	RCdB	0.053	0.003	0.004	LCdU	0.080	0.017	0.03
	RHgU	0.215	0.001	0.03	RHgU	0.216	0.0003	0.02
	LCreatU	–0.665	0.0861	< 0.001	LCreatU	–0.593	0.076	< 0.001
	LPbB × RHgU	–0.175	0.0145	0.01	LPbB × RHgU	–0.161	0.012	0.03
	RHgU × RCdB	0.069	0.0197	0.01				
RHVAU	LCreatU	–1.626	0.0867	< 0.001	LCdU	0.183	0.0152	0.02
	LPbB × RHgU	–0.083	0.0129	0.02	LCreatU	–1.489	0.0776	< 0.001
	RHgU × RCdB	0.194	0.0262	< 0.001	LPbB × RHgU	–0.065	0.0095	0.03

Variables preceded by L have been log-transformed; those preceded by R are ranked variables.
